# Nanomolar Inhibitors of *Trypanosoma brucei* RNA Triphosphatase

**DOI:** 10.1128/mBio.00058-16

**Published:** 2016-02-23

**Authors:** Paul Smith, C. Kiong Ho, Yuko Takagi, Hakim Djaballah, Stewart Shuman

**Affiliations:** a Molecular Biology Program, Sloan-Kettering Institute, New York, New York, USA; b Department of Infection Biology, Faculty of Medicine, University of Tsukuba, Tsukuba, Ibaraki, Japan; c Department of Biological Sciences, State University of New York at Buffalo, Buffalo, New York, USA; d High Throughput Screening Core Facility, Memorial Sloan-Kettering Cancer Center, New York, New York, USA

## Abstract

Eukaryal taxa differ with respect to the structure and mechanism of the RNA triphosphatase (RTPase) component of the mRNA capping apparatus. Protozoa, fungi, and certain DNA viruses have a metal-dependent RTPase that belongs to the triphosphate tunnel metalloenzyme (TTM) superfamily. Because the structures, active sites, and chemical mechanisms of the TTM-type RTPases differ from those of mammalian RTPases, the TTM RTPases are potential targets for antiprotozoal, antifungal, and antiviral drug discovery. Here, we employed RNA interference (RNAi) knockdown methods to show that *Trypanosoma brucei* RTPase Cet1 (TbCet1) is necessary for proliferation of procyclic cells in culture. We then conducted a high-throughput biochemical screen for small-molecule inhibitors of the phosphohydrolase activity of TbCet1. We identified several classes of chemicals—including chlorogenic acids, phenolic glycopyranosides, flavonoids, and other phenolics—that inhibit TbCet1 with nanomolar to low-micromolar 50% inhibitory concentrations (IC_50_s). We confirmed the activity of these compounds, and tested various analogs thereof, by direct manual assays of TbCet1 phosphohydrolase activity. The most potent nanomolar inhibitors included tetracaffeoylquinic acid, 5-galloylgalloylquinic acid, pentagalloylglucose, rosmarinic acid, and miquelianin. TbCet1 inhibitors were less active (or inactive) against the orthologous TTM-type RTPases of mimivirus, baculovirus, and budding yeast (*Saccharomyces cerevisiae*). Our results affirm that a TTM RTPase is subject to potent inhibition by small molecules, with the caveat that parallel screens against TTM RTPases from multiple different pathogens may be required to fully probe the chemical space of TTM inhibition.

## IMPORTANCE

The stark differences between the structure and mechanism of the RNA triphosphatase (RTPase) component of the mRNA capping apparatus in pathogenic protozoa, fungi, and viruses and those of their metazoan hosts highlight RTPase as a target for anti-infective drug discovery. Protozoan, fungal, and DNA virus RTPases belong to the triphosphate tunnel metalloenzyme family. This study shows that a protozoan RTPase, TbCet1 from *Trypanosoma brucei*, is essential for growth of the parasite in culture and identifies, via *in vitro* screening of chemical libraries, several classes of potent small-molecule inhibitors of TbCet1 phosphohydrolase activity.

## INTRODUCTION

The m^7^G cap structure of eukaryal mRNA promotes translation initiation and protects mRNA from degradation by 5′ exoribonucleases. All eukaryal species and many eukaryal viruses share a “classic” three-step capping pathway in which (i) an RNA triphosphatase (RTPase) removes the γ-phosphate of the primary transcript, (ii) an RNA guanylyltransferase transfers GMP from GTP to the 5′-diphosphate RNA to form a GpppRNA cap, and (iii) a cap-specific RNA (guanine-N7) methyltransferase adds a methyl group from *S*-adenosylmethionine (AdoMet) to the cap guanine to form the m^7^GpppRNA structure (1).

Eukaryal taxa differ with respect to the structure and mechanism of the RTPase component of the capping apparatus. Fungi and protozoa have a metal-dependent RTPase that belongs to the triphosphate tunnel metalloenzyme (TTM) superfamily (2, 3). The crystal structure of the *Saccharomyces cerevisiae* RTPase Cet1 (SceCet1) (4) revealed a novel fold in which the active site is located in the center of a topologically closed 8-stranded antiparallel β-barrel (the triphosphate tunnel). The TTM active site is composed of essential amino acids that either coordinate a metal ion or the γ-phosphate or stabilize the tunnel architecture (4–7). Biochemical characterization, comparative mutational analyses, and/or structure determinations by X-ray crystallography have shown that the RTPases of fungi *Candida albicans* and *Schizosaccharomyces pombe* (8–10); protozoan parasites *Trypanosoma brucei* (11, 12) *Plasmodium falciparum* (3, 13), *Encephalitozoon cuniculi* (14), and *Giardia lamblia* (15); and DNA viruses vaccinia virus (16, 17), mimivirus (18), baculovirus (19–22), and *Chlorella* virus *Paramecium bursaria Chlorella* virus 1 (PBCV-1) (23, 24) all belong to the TTM superfamily. In contrast, metazoan and plant RTPases are metal-independent enzymes of the cysteine-phosphatase superfamily (25, 26), and they catalyze γ-phosphate hydrolysis via a covalent protein-cysteinyl-*S*-phosphoester intermediate (26).

The fact that the tertiary structures, active sites, and chemical mechanisms of the TTM-type RTPases are completely different from those of mammalian cysteine-phosphatase-type RTPases highlights TTM RTPases as promising targets for antifungal, antiprotozoal, and antiviral drug discovery predicated on interdicting the capping of the pathogen’s mRNAs while sparing the host’s capping pathway (27). The TTM RTPases Cet1, Pct1, and TriA are essential for the growth of the budding yeast *S. cerevisiae*, the fission yeast *S. pombe*, and the human-pathogenic fungus *Aspergillus fumigatus*, respectively (5, 28, 29). To our knowledge, it has not been determined whether a TTM RTPase is essential for a protozoon. Here, we employed RNA interference (RNAi) to show that *T. brucei* RTPase Cet1 (TbCet1) is necessary for proliferation of procyclic cells in culture.

Yeast *ceg1*Δ strains that rely for viability on the activity of either fungal, viral, and protozoan TTM RTPases or mammalian cysteine-phosphatase RTPases afford enabling tools for cell-based screens and counterscreens for cytotoxins that inhibit TTM RTPases (11, 30, 31). *In vitro* screening for inhibitors of TTM RTPases is simplified by their signature biochemical property of hydrolyzing nucleoside triphosphates (NTPs) to nucleoside diphosphates (NDPs) and inorganic phosphate (P_i_) in the presence of manganese (2), thereby avoiding the need to prepare triphosphate-terminated RNAs as the substrates.

In the present study, we conducted a biochemical screen for small-molecule inhibitors of the *T. brucei* RTPase TbCet1. Kinetoplastid protozoan parasites of the genus *Trypanosoma* are major zoonotic pathogens of humans. *Trypanosoma cruzi* is the cause of Chagas disease, endemic in South America. *T. brucei*, transmitted by tsetse flies, causes sleeping sickness in sub-Saharan Africa. The drugs used to treat trypanosomiasis (pentamidine, suramin, melarsoprol, and eflornithine) either are old, have many undesirable side effects, are not effective against late-stage disease, or are cumbersome to administer (32). There is a need for new therapeutic approaches.

TbCet1 is a 252-amino-acid (aa) monomeric TTM protein that can function in cap formation *in vivo* in *S. cerevisiae* in lieu of yeast Cet1 (11, 12). Recombinant TbCet1 has vigorous manganese-dependent ATPase activity (*k*_cat_, 59 s^−1^) that is effaced by alanine mutations of conserved active site residues in the triphosphate tunnel. TbCet1 is exceptionally thermostable. Tripolyphosphate is a potent competitive inhibitor of TbCet1 (*K_i_*, 1.4 µM) that binds more avidly to the active site than the ATP substrate (*K_m_*, 25 µM). Synergistic activation of the TbCet1 triphosphatase by manganese and magnesium suggested a two-metal mechanism of catalysis (12). Recent crystallographic studies of other TTM family members indicate that two-metal catalysis is a core feature of the TTM clade (33).

Here, we implemented a colorimetric assay of phosphate release from ATP by TbCet1 for the purpose of high-throughput screening (HTS) for small-molecule inhibitors. The initial questions were (i) can potent TbCet1 inhibitors be identified, and (ii) if so, do they illuminate common features? The answer to both questions is yes, as described below. Part of the allure of TTM RTPases as anti-infective drug targets is the prospect of obtaining an inhibitor with broad-spectrum potency against fungal, protozoal, and viral RTPases. To put this idea to the test, we surveyed the newly identified inhibitors of TbCet1 for their effects on exemplary fungal and viral TTM RTPases and on a non-RTPase bacterial TTM enzyme (34, 35). The results indicated that the inhibitors generally displayed greatest potency and selectivity for the TbCet1 enzyme against which they were screened, signifying that parallel screening against TTM RTPases from pathogens of interest may be required to best explore the chemical space of TTM inhibition.

## RESULTS

### Inducible RNAi knockdown of TbCet1 arrests the growth of *T. brucei* in culture.

**FIG 1:**
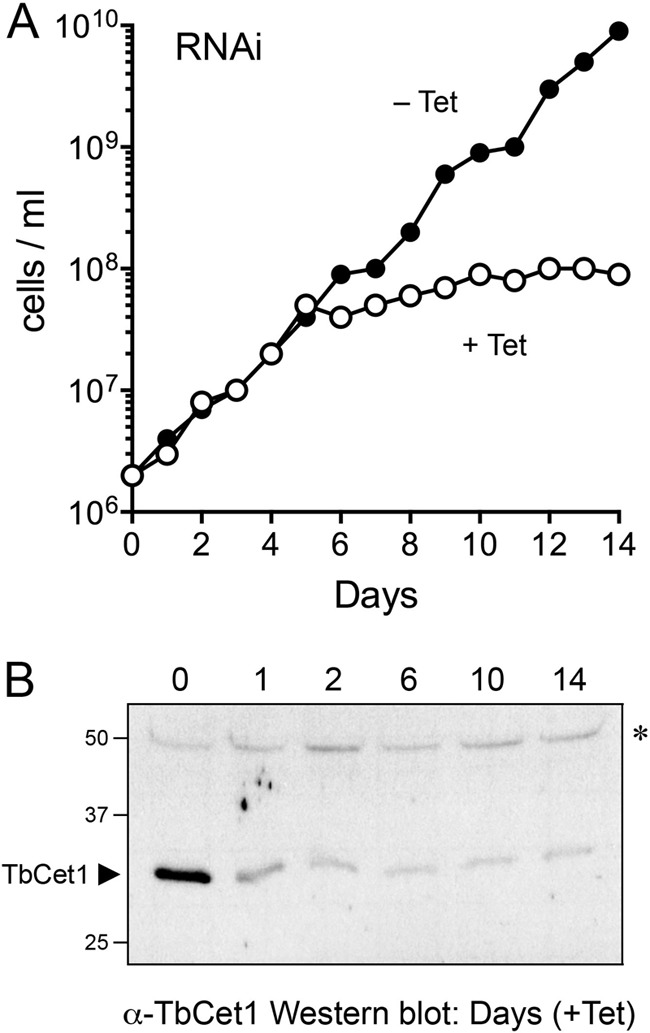
RNAi knockdown of TbCet1 arrests growth of *T. brucei*. (A) Procyclic *T. brucei* 29.13-TbCet1RNAi transfectants were inoculated into medium containing 1.0 µg/ml tetracycline (+Tet) to induce TbCet1 dsRNA production and into a parallel control culture lacking tetracycline (−Tet). Cell density was monitored by microscopy and maintained between 1 × 10^6^ and 1 × 10^7^ cells/ml by dilution into fresh medium. The growth curves display on the *y* axis the log of the direct cell count multiplied by the dilution factor. Induction of RNAi against TbCet1 arrested *T. brucei* growth after 5 days. (B) Western blotting verifies knockdown of TbCet1 protein by RNAi induction. An anti-TbCet1 immunoblot assay of total protein from cells harvested from a +Tet culture is shown. The positions and sizes (kilodaltons) of marker polypeptides are indicated on the left. The immunoreactive TbCet1 polypeptide, denoted by the arrowhead at left, is depleted after RNAi induction. A nonspecific cross-reacting 50-Da polypeptide, indicated by the asterisk at right, is unchanged.

TbCet1 was depleted in *T. brucei* procyclic cells by using an RNAi system in which the synthesis of double-stranded TbCet1 RNA by T7 RNA polymerase is tetracycline inducible. In the experiment shown in Fig. 1A, the cells were inoculated into medium containing 1.0 µg/ml tetracycline (+Tet) to induce TbCet1 double-stranded RNA (dsRNA) production and into a parallel control culture lacking tetracycline (−Tet). The control uninduced cells maintained logarithmic growth over a 14-day period. In contrast, the induction of TbCet1 RNAi by Tet acutely curtailed cell proliferation after an initial 5-day interval of logarithmic growth. RNAi depletion of cellular TbCet1 protein was verified by Western blot analysis of total protein from +Tet cells (Fig. 1B). The level of TbCet1 protein was serially decreased to 26% of the initial value after 1 day of incubation in tetracycline, to 12% after 2 days, and to ≤8% for the rest of the 14-day period of RNAi induction. These results indicate that TbCet1 is essential for *T. brucei* growth, and they encourage screening for TbCet1 inhibitors as potential antitrypanosomal drugs.

### Test library screening for inhibitors of TbCet1 ATPase.

We applied a colorimetric assay using malachite green reagent to gauge the release of inorganic phosphate from ATP by recombinant TbCet1 in the presence of manganese. The Z′ score for this assay was 0.88. Z′ is a measure of the signal-to-noise ratio intrinsic to an experiment and is calculated by comparing the typical variance in values for positive and negative controls with the dynamic range seen between the two. Values above 0.5 indicate that an assay is well suited for high-throughput screening (36).

**FIG 2:**
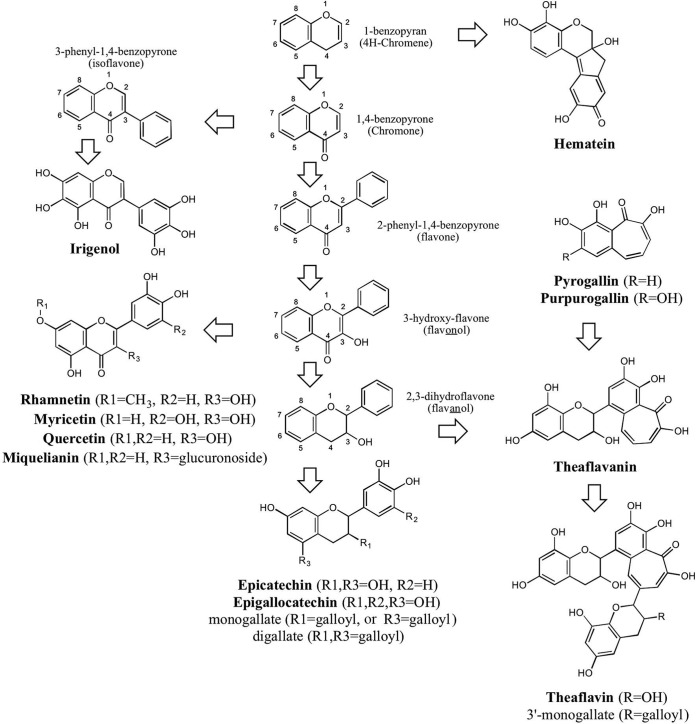
Chemical structures of flavonoid TbCet1 inhibitors and related compounds. Core scaffolds of flavonoids are labeled in lightface. TbCet1 inhibitors identified in the present study are labeled in boldface (with R groups in parentheses as specified). Arrows signify progressive chemical modifications.

The susceptibility of TbCet1 to chemical inhibition was tested via an initial screen of a library of 2,879 structurally diverse compounds comprising pharmaceuticals, natural products, and industrial chemicals. We identified 22 compounds from this collection that reproducibly inhibited the TbCet1 ATPase activity by ≥75% at a 10 µM concentration (Table 1). Nineteen of the 22 inhibitors were phenolic compounds that fell into discrete chemical classes, either flavonoids (11/22), phenolic glucopyranosides (2/22), or nonflavonoid phenolics (6/22). The chemical structures of the flavonoids and selected phenolics are shown in Fig. 2. In a previous study (37), a screen of the same test library for TbCet1 ATPase inhibitors using a fluorescence polarization-based assay (Transcreener) for detection of ADP formation yielded 23 confirmed positives with 50% inhibitory concentrations (IC_50_s) of less than 10 µM. Sixteen of the TbCet1 inhibitors identified in the present screen using a phosphate release assay were identified independently by assaying inhibition of ADP release.

**TABLE 1:** TbCet1 inhibitors identified in the initial test screen of a 2,879-compound library

Class	Name
Flavonoid	Irigenol
	Irigenol hexaacetate
	Myricetin
	Epigallocatechin-3-monogallate
	2′,2′-Bis-epigallocatechin monogallate
	Epigallocatechin digallate
	Epicatechin monogallate
	Theaflavin monogallate
	Theaflavanin
	Rhamnetin
	Quercetin
Phenolic	Pyrogallin
	Purpurogallin
	Ellagic acid
	Hematein
	Norepinephrine
	Chicago sky blue 6B
Phenolic glucopyranoside	Tannic acid
	Sennoside A
Other	Merbromin
	*N*-Hydroxymethyl-nicotinamide
	Gangleoidin acetate

### HTS for TbCet1 inhibitors.

**FIG 3:**
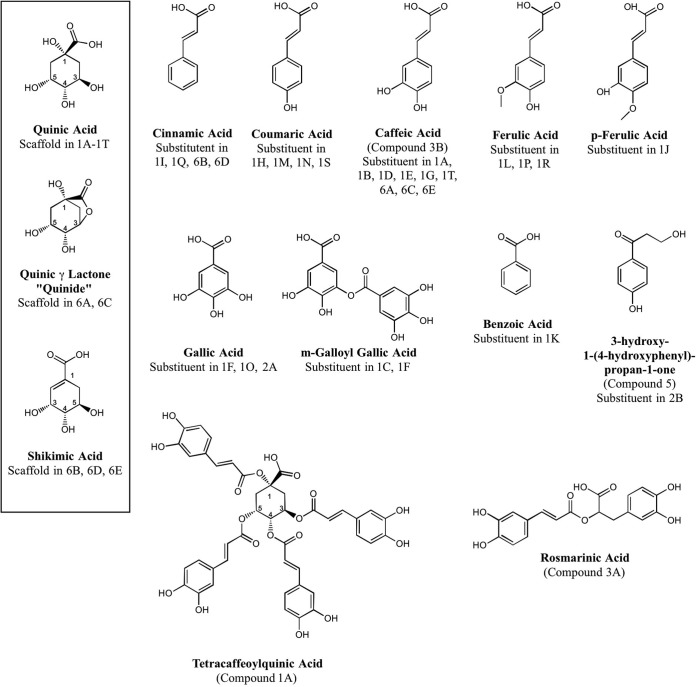
Chemical structures of chlorogenic acid TbCet1 inhibitors and related compounds. Core scaffolds of quinic acid, quinide, and shikimic acid are depicted in the box at left. Nine substituents esterified to the scaffolds are shown in the top and middle rows. The structures of tetracaffeoylquinic acid and rosmarinic acid are shown at bottom.

We next applied the colorimetric high-throughput screening (HTS) assay to survey 191,050 compounds sourced from four different commercial libraries, of which 543 (0.28%) inhibited the ATPase activity of TbCet1 by at least 50% at a 10 µM concentration. A set of 216 nonredundant compounds were resupplied by the vendors, 83 (38%) of which showed reproducible inhibition of TbCet1 upon retesting and were thus designated confirmed “hit” compounds. The hit compounds were then subjected to IC_50_ determination via the HTS assay. Nine compounds had IC_50_s of 1.1 µM or less (Table 2). These were, in decreasing order of potency, 1,3,4,5-*O*-tetracaffeoylquinic acid (compound 1A; IC_50_, 20 nM); 3,5-*O*-dicaffeoylquinic acid (compound 1B; IC_50_, 40 nM); pentagalloylglucose (compound 2A, IC_50_, 40 nM); rosmarinic acid (compound 3A; IC_50_, 50 nM); three flavonoids, miquelianin (compound 4A; IC_50_, 180 nM), myricetin (compound 4B; IC_50_, 230 nM), and epicatechin (compound 4C; IC_50_, 260 nM); 3-hydroxy-1-(4-hydroxyphenyl)propan-1-one (compound 5; IC_50_, 630 nM); and 3-*O*-(4-hydroxyphenylpropanone)-1-glucopyranoside (compound 2B; IC_50_, 1.1 µM). Chemical structures of the constituents of these compounds are shown in Fig. 2 and 3.

**TABLE 2:** Submicromolar TbCet1 inhibitors identified via HTS and confirmed by manual assay

Compound	Name	Class	IC_50_ (µM) by assay:	
			HTS	^32^P
1A	Tetracaffeoylquinic acid	Quinic acid ester	0.02	0.013
1B	3,5-Dicaffeoylquinic acid	Quinic acid ester	0.04	0.07
2A	Pentagalloylglucose	Glucopyranoside	0.04	0.046
2B	3-*O*-(4-Hydroxyphenylpropanone)-1-glucopyranoside	Glucopyranoside	1.1	0.66
3A	Rosmarinic acid	Phenolic	0.05	0.12
4A	Miquelianin	Flavonoid	0.18	0.18
4B	Myricetin	Flavonoid	0.23	0.36
4C	Epicatechin	Flavonoid	0.26	0.25
5	3-Hydroxy-1-(4-hydroxyphenyl)propan-1-one	Phenolic	0.63	0.64

**FIG 4:**
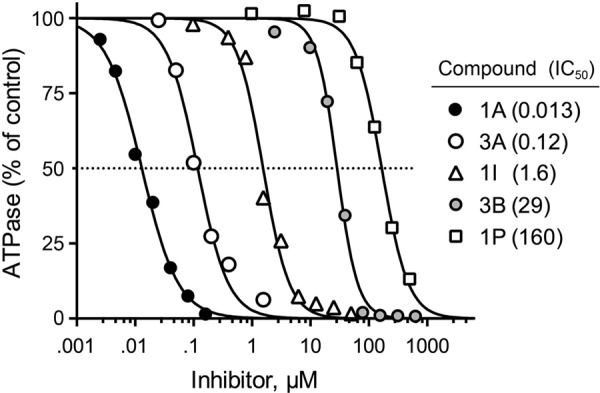
Potency spectrum of TbCet1 inhibitors. Dose-response profiles for tetracaffeoylquinic acid (1A), rosmarinic acid (3A), tetracinnamoylquinic acid (3I), caffeic acid (3B), and 3-*O*-coumeroylquinic acid (1P). IC_50_s (µM) are indicated in parentheses.

Each of these nine compounds was then retested for TbCet1 inhibition via a direct assay of the release of ^32^P_i_ from [γ-^32^P]ATP, in which the reaction products were analyzed by polyethyleneimine (PEI)-cellulose thin-layer chromatography (TLC). Logarithmic plots of inhibition versus compound concentration are shown in Fig. 4 for tetracaffeoylquinic acid (compound 1A; IC_50_, 13 nM) and rosmarinic acid (compound 3A; IC_50_, 120 nM), as well as for three less potent compounds (to be discussed below). The IC_50_s for the radiolabeled ATP hydrolysis assay agreed fairly well with those from the colorimetric phosphate release assay employed for HTS (Table 2). In the radioactive phosphate release assay, the TbCet1, ATP, and Mn^2+^ concentrations were 2.2 nM, 0.2 mM, and 2 mM, respectively. Thus, the nanomolar to micromolar inhibition observed for the 9 hit compounds cannot be attributable to sequestration of metal cofactor or nucleotide substrate. The direct radiolabel phosphate release assay was employed for all further studies.

### Inhibition of TbCet1 by chlorogenic acids and their derivatives.

Chlorogenic acids are compounds wherein cinnamic acid or one of its derivatives is esterified to a quinic acid scaffold (Fig. 3). Chlorogenic acids often contain multiple ester substitutions on the quinic acid hydroxyl groups. The two most potent inhibitors of TbCet1 identified by HTS were the chlorogenic acids tetracaffeoylquinic acid (compound 1A) and 3,5-dicaffeoylquinic acid (compound 1B). Caffeic acid is the dihydroxy derivative of cinnamic acid (Fig. 3). To ascertain the inhibitory properties of compounds similar to these, we screened an additional 14 chlorogenic acids and 4 structurally related quinic acid esters for inhibition of TbCet1 (compounds 1C to 1T [Table 3]). Four of these compounds (1D, 1E, 1G, and 1T) contained caffeic acid substituents. Two compounds (1I and 1Q) contained cinnamic acid. Eight others (1H, 1J, 1L, 1M, 1N, 1P, 1R, and 1S) included three different derivatives of cinnamic acid: coumaric acid, ferulic acid, and *p*-ferulic acid (chemical structures shown in Fig. 3). Coumaric acid differs from caffeic acid by the removal of the *meta* hydroxyl moiety. Ferulic acid and *p*-ferulic acid are O-methyl derivatives of caffeic acid at the *meta* and *para* positions, respectively (Fig. 3). Though not chlorogenic acids, the 4 additional quinic acid esters had related phenolic substituents in place of a cinnamic acid derivative. These were gallic acid (3,4,5-trihydroxybenzoic acid [Fig. 3]) in compounds 1F and 1O; *m*-galloyl gallic acid (a dimer of gallic acid [Fig. 3]) in compounds 1C and 1F; and benzoic acid in compound 1K. Whereas none of these 18 compounds were better inhibitors than tetracaffeoylquinic acid (compound 1A) and 3,5-dicaffeoylquinic acid (compound 1B), five of the quinic acid esters displayed IC_50_s below 1 µM (compounds 1C, 1D, 1E, 1F, and 1G). These were the quinic acid monoester to *m*-galloylgallic acid (at position R-5; IC_50_, 110 nM), 3,4,5- and 1,3,5-triesters to caffeic acid (IC_50_s, 150 nM and 190 nM, respectively), and mixed 3-galloyl,5-galloylgalloylquinic acid diester (IC_50_, 220 nM). Four of the quinic acid esters had IC_50_s between 1 and 10 µM: the 3,4-dicoumarolyl compound 1H (IC_50_, 1.3 µM); the 1,3,4,5-tetracinnamoyl compound 1I (IC_50_, 1.6 µM [Fig. 4]); the 5-*p*-feruloyl compound 1J (IC_50_, 2.4 µM); and the 1,3,4,5-tetrabenzoyl compound 1K (IC_50_, 5.8 µM).

**TABLE 3:** Quinic acid esters tested for inhibition of TbCet1

Compound	R1	R3	R4	R5	IC_50_ (µM)
1A	Caffeic acid	Caffeic acid	Caffeic acid	Caffeic acid	0.013
1B		Caffeic acid		Caffeic acid	0.07
1C				*m*-Galloyl-gallic acid	0.11
1D		Caffeic acid	Caffeic acid	Caffeic acid	0.15
1E	Caffeic acid	Caffeic acid		Caffeic acid	0.19
1F		Gallic acid		*m*-Galloyl-gallic acid	0.22
1G	Caffeic acid	Caffeic acid			0.34
1H		Coumaric acid	Coumaric acid		1.3
1I	Cinnamic acid	Cinnamic acid	Cinnamic acid	Cinnamic acid	1.6
1J				*p*-Ferulic acid	2.4
1K	Benzoic acid	Benzoic acid	Benzoic acid	Benzoic acid	5.8
1L				Ferulic acid	12
1M		Coumaric acid		Coumaric acid	24
1N			Coumaric acid		25
1O				Gallic acid	125
1P		Ferulic acid			160
1Q				Cinnamic acid	210
1R	Ferulic acid				>500
1S		Coumaric acid			>500
1T				Caffeic acid	>500

Rosmarinic acid (compound 3A) resembles a chlorogenic acid but contains no central scaffold analogous to quinic acid; rather, rosmarinic acid can be considered a partially hydrogenated dimer of caffeic acid. Whereas rosmarinic acid inhibited TbCet1 with an IC_50_ of 120 nM, a simple caffeic acid monomer (compound 3B) had an IC_50_ of 29 µM (Fig. 4). Quinic acid showed no inhibition of TbCet1, nor did gallic acid.

### Structure-activity relationships for chlorogenic acids.

The importance of both the size of the quinic acid ester substituents and their content of hydroxyl groups is underscored by comparing 5-galloylquinic acid (compound 1O; IC_50_, 125 µM) with 5-galloyl-galloylquinic acid (compound 1C; IC_50_, 110 nM), whereby the latter is 1,100-fold more potent than the former as a TbCet1 inhibitor. Correlation between substituent size and hydroxyl content is also evident when all hydroxy groups are subtracted from the most potent inhibitor, tetracaffeoylquinic acid (compound 1A; IC_50_, 13 nM), to yield the 120-fold-less-potent tetracinnamoylquinic acid (compound 1I; IC_50_, 1.6 µM). This theme is fortified by comparing 3,5-dicaffeoylquinic acid (compound 1B; IC_50_, 70 nM) with 3,5-dicoumaroylquinic acid (compound 1M; IC_50_, 24 µM), whereby the subtraction of one *meta* hydroxyl group on each of the esterified substituents results in a 340-fold drop in the potency of TbCet1 inhibition.

Hydroxyl content *per se* is not predictive of potency for the 5-O-monoesters of quinic acid. In this series, a 5-caffeoyl group in compound 1T (2 hydroxyls) conferred no activity (IC_50_, >500 µM), and a 5-galloyl moiety in compound 1M (3 hydroxyls) and a 5-cinnamoyl group (no hydroxyls) in compound 1Q conferred similarly feeble inhibition (IC_50_, 125 to 210 µM), whereas 5-feruoylquinic acid (compound 1L; IC_50_, 12 µM) and 5-*p*-feruoylquinic acid (compound 1J; IC_50_, 2.4 µM) were much more potent. The comparison of inactive 5-caffeoylquinic acid with active 5-feruoyl and 5-feruoyl derivatives illustrates the salutary effect of a methoxy group versus a hydroxyl at the *meta* or *para* positions. The position of monoesterification to the quinic acid scaffold is highlighted as a governor of inhibitor potency in the series of feruoylquinic acid isomers, whereby 4-feruoylquinic acid (compound 1P; IC_50_, 160 µM) is 13-fold less effective than 5-feruoylquinic acid and 1-feruoylquinic acid (compound 1R) is inactive.

### Test of inhibition by quinide and shikimic acid esters.

Quinic acid can be derivatized to a gamma lactone by formation of a cyclic ester of the 1-carboxylic acid and 3-hydroxy groups to give a 1-3 quinic acid lactone, colloquially dubbed a “quinide.” (Controversy exists as to the nomenclature of the resulting lactone [shown in Fig. 3], but the convention employed here employs the “*cis* before *trans*” rule of the Cahn-Ingold-Prelog naming system, and thus, the lactone under consideration is termed a 1-3 lactone, not a 1-5 lactone.) The 1,4,5-tricaffeoyl derivative of quinide (compound 6A [Table 4]) was a potent inhibitor of TbCet1 (IC_50_, 120 nM), similar to the tricaffeoyl esters of quinic acid (compounds 1D and 1E [Table 3]). Substituting acetate for the R1 caffeic acid in the quinide ester (compound 6C) lowered potency by 30-fold (IC_50_, 3.6 µM). Quinic acid can also undergo a dehydration reaction at C-1 to form shikimic acid (Fig. 3). We found that 3,4,5-tricinnamoylshikimic acid (compound 6B) inhibited TbCet1 with an IC_50_ of 1.5 µM (Table 4), similar to the IC_50_ of 1.6 µM for the tetracinnamoyl ester of quinic acid (compound 1I [Fig. 3; Table 3]). The derivatives 3,5-dicannamolylshikimate (compound 6D) and 5-caffeoylshikimate (compound 6E) did not inhibit TbCet1 (Table 4).

**TABLE 4:** Quinide and shikimate esters tested for inhibition of TbCet1

Compound	Modification	R1	R3	R4	R5	IC_50_ (µM)
6A	Quinide	Caffeic acid		Caffeic acid	Caffeic acid	0.12
6B	Shikimate		Cinnamic acid	Cinnamic acid	Cinnamic acid	1.5
6C	Quinide	Acetate		Caffeic acid	Caffeic acid	3.6
6D	Shikimate		Cinnamic acid		Cinnamic acid	>500
6E	Shikimate				Caffeic acid	>500

### Selectivity of TbCet1 inhibitors.

The promise of TTM RTPases as anti-infective drug targets would be greatest if one could identify inhibitors with broad-spectrum potency against fungal, protozoal, and viral TTM RTPases. To see if this property applied to any of the submicromolar TbCet1 inhibitors identified currently, we surveyed their effects on (i) *S. cerevisiae* Cet1; (ii) the RNA triphosphatase component of mimivirus capping enzyme (MimiCE) (18); (iii) the RNA triphosphatase component of the baculovirus *Autographa californica* nucleopolyhedrovirus (AcNPV) capping enzyme Lef4 (20–22); and (iv) *Clostridium thermocellum* TTM (CthTTM), a bacterial TTM family member that hydrolyzes inorganic polyphosphates, NTPs, and dinucleotide polyphosphate substrates (34, 35). The IC_50_s of 25 compounds against the five ATPase enzymes surveyed are compiled in Table 5.

**TABLE 5:** Selectivity of inhibition of TbCet1 versus other TTM triphosphatases

Compound	IC_50_, µM (IC_50_/IC_50_ TbCet1)				
	TbCet1	MimiCE	SceCet1	CthTTM	Lef4
1A	0.013	1.4 (108)	7.1 (546)	11 (846)	5.7 (438)
1C	0.11	>500	>500	>500	75 (682)
1D	0.15	16 (107)	10 (67)	160 (1,067)	7.2 (48)
1E	0.19	8.4 (44)	29 (153)	33 (174)	5.6 (29)
1F	0.22	3.4 (15)	>500	21 (95)	33 (150)
1G	0.34	3.7 (11)	>500	55 (162)	22 (65)
1H	1.3	52 (40)	>500	>500	140 (108)
1I	1.6	17 (11)	>500	17 (11)	6 (4)
1J	2.4	>500	>500	>500	>500
1K	5.8	20 (3)	>500	72 (12)	46 (8)
1L	12	>500	>500	>500	>500
1M	24	>500	>500	>500	>500
1N	25	>500	>500	>500	>500
1O	125	>500	10 (0.08)	>500	>500
2A	0.046	5.1 (111)	3.2 (70)	1.4 (30)	59 (1,283)
2B	0.66	120 (182)	41 (62)	29 (44)	150 (227)
3A	0.12	4.3 (36)	3.3 (28)	1.5 (13)	3.2 (27)
3B	29	>500	>500	310 (11)	75 (3)
4A	0.18	19 (106)	43 (239)	24 (133)	29 (161)
4B	0.36	20 (56)	26 (72)	6.5 (18)	160 (444)
4C	0.25	10 (40)	8.7 (35)	26 (104)	34 (136)
5	0.64	84 (131)	>500	9.2 (14)	160 (250)
6A	0.12	2 (17)	2 (17)	6.4 (53)	2.6 (22)
6B	1.5	19 (13)	>500	150 (100)	10 (7)
6C	3.6	15 (4)	14 (4)	46 (13)	21 (6)

**FIG 5:**
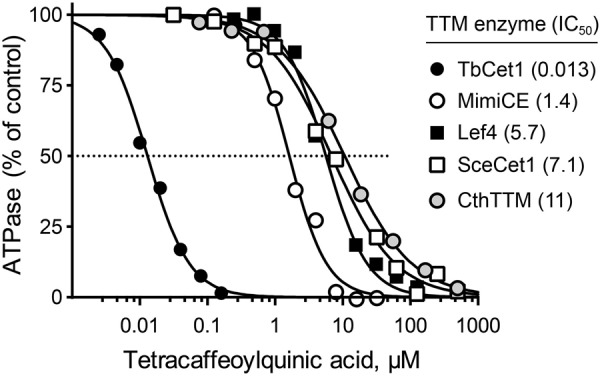
Tetracaffeoylquinic acid activity against five TTM family ATPases. Dose-response profiles for tetracaffeoylquinic acid inhibition of the indicated TTM enzymes. IC_50_s (µM) are indicated in parentheses.

Tetracaffeoylquinic acid (compound 1A), the most potent of the TbCet1 inhibitors (IC_50_. 13 nM), cross-inhibited all of the other enzymes (Fig. 5), albeit with much lower potency in the following hierarchy: 110-fold lower against MimiCE (1.4 µM), 440-fold lower against Lef4 (5.7 µM), 550-fold lower against SceCet1 (7.1 µM), and 850-fold lower against CthTTM (11 µM). 3,4,5-Tricaffeoylquinic acid (compound 1D; IC_50_, 150 nM for TbCet1) cross-inhibited with similar trends: 48-fold lower against Lef4 (7.2 µM), 67-fold lower against SceCet1 (10 µM), 107-fold lower against MimiCE (16 µM), and 1,100-fold lower against CthTTM (160 µM). Galloyl-galloylquinic acid (compound 1C), with an IC_50_ of 110 nM versus TbCet1, was inactive against MimiCE, SceCet1, and CthTTM (IC_50_, >500 µM) and only weakly active against Lef4 (IC_50_, 75 µM; i.e., 680-fold less active compared with its effect on TbCet1). Thus, these three quinic acid ester compounds are quite specific for TbCet1 among the TTM enzyme family enzymes tested. Similar trends are seen for other quinic acid ester inhibitors of TbCet1: 1,3,5-tricaffeoylquinic acid (compound 1E), 3-galloyl,5-galloyl-galloylquinic acid (compound 1F), 1,3-dicaffeoylquinic acid (compound 1G), 3,4-dicoumaroylquinic acid (compound 1H), and others in Table 5. An interesting exception was 5-galloylquinic acid (compound 1O), which was a very weak TbCet1 inhibitor (IC_50_, 125 µM) but emerged as a selective and modestly potent SceCet1 inhibitor (IC_50_, 10 µM) that had no activity against MimiCE, Lef4, or CthTTM (Table 5).

Of the two quinide ester TbCet1 inhibitors, the more potent 1,4,5-tricaffeoylquinide (compound 6A; IC_50_, 120 nM) was 110- to 240-fold less effective against other TTM enzymes. The less potent 1-acetyl,4,5-dicaffeoylquinide (compound 6C; IC_50_, 3.6 µM) was also the least specific, with IC_50_s ranging from just 4-fold to 13-fold greater against the four other TTM ATPases. Tricinnamoylshikimate (compound 6B), with an IC_50_ of 1.5 µM versus TbCet1, was 7- to 13-fold less active against Lef4 and MimiCE, 100-fold less active against CthTTM, and inactive against SceCet1.

Pentagalloylglucose (compound 2A) was the second most potent antagonist of TbCet1 identified via our HTS screen (IC_50_, 46 nM). (A related glucopyranoside tannic acid [pentagalloylgalloylglucose] was identified as a TbCet1 inhibitor [IC_50_, 60 nM] in the small-scale test library screens performed here and previously [37].) Pentagalloylglucose inhibited the other enzymes with much lower potency in the following order: 30-fold lower against CthTTM (1.4 µM), 70-fold lower against SceCet1 (3.2 µM), 110-fold lower against MimiCE (5.1 µM), and 1,300-fold lower against Lef4 (150 µM). 3-*O*-(4-Hydroxyphenylpropanone)-1-glucopyranoside (compound 2B), which inhibited TbCet1 with an IC_50_ of 660 nM, has a cross-inhibitory spectrum similar to that of pentagalloylglucose against three of the other enzymes: 44-fold lower against CthTTM (29 µM), 62-fold lower against SceCet1 (41 µM), and 180-fold lower versus MimiCE (120 µM). It is noteworthy that 3-hydroxy-1-(4-hydroxyphenyl)propan-1-one itself (compound 5), which was as effective as 3-*O*-(4-hydroxyphenylpropanone)-1-glucopyranoside versus TbCet1 (IC_50_, 640 nM), differed from the glucopyranoside in that 3-hydroxy-1-(4-hydroxyphenyl)propan-1-one had no effect on SceCet1.

Rosmarinic acid (compound 3A), with an IC_50_ of 120 nM versus TbCet1, inhibited the other enzymes tested with IC_50_s ranging from 1.5 to 4.3 µM (i.e., 13- to 36-fold less than against TbCet1). Caffeic acid monomer (compound 3B), which weakly inhibited TbCet1 (IC_50_, 29 µM) and Lef4 (IC_50_, 75 µM), was even less active against CthTTM (310 µM) and inactive against MimiCE and SceCet1.

The flavonoid inhibitors of TbCet1 miquelianin (compound 4A; IC_50_, 180 nM), myricetin (compound 4B; IC_50_, 360 nM), and epicatechin (compound 4C; IC_50_, 250 nM) were 40- to 110-fold less active against MimiCE, 35- to 240-fold less active against SceCet1, 18- to 26-fold less potent against CthTTM, and 140- to 440-fold less effective against Lef4.

## DISCUSSION

The present study affirms that a protozoan TTM RTPase, TbCet1 from *T. brucei*, is essential for growth of the parasite in culture and is subject to inhibition *in vitro* by small molecules at low-micromolar and nanomolar concentrations. Applying HTS methods to an in-house library of nearly 200,000 compounds, we have identified several classes of chemicals (including chlorogenic acids, phenolic glycopyranosides, flavonoids, and other phenolics) that inhibit TbCet1. We subsequently confirmed the activity of the HTS hits, and various analogs thereof, by direct manual assays of phosphohydrolase activity.

The majority of the TbCet1 inhibitors that we identified showed strong specificity for the trypanosome enzyme and were manyfold less active (or inactive in some cases) against the orthologous TTM-type RTPases of mimivirus, baculovirus, and budding yeast (or the homologous bacterial phosphohydrolase CthTTM). Thus, (i) potent inhibition of a particular RTPase is no guarantee that the inhibitory compounds will have a quantitatively similar effect on another member of the TTM family, and (ii) an optimal search for inhibitors of the TTM-type mRNA capping enzymes of infectious pathogens will entail screening against RTPases from each organism or virus of interest.

From the data in Table 5, we can infer certain trends in the responsiveness of other TTM enzymes to TbCet1 inhibitors. For example, SceCet1 was the least responsive, insofar as 13/25 TbCet1 inhibitors had no effect on the yeast enzyme (IC_50_, >500 µM). MimiCE was unaffected by 7/25 inhibitors. CthTTM was also unaffected by 7/25 inhibitors, six of which overlapped with the compounds inactive against MimiCE. Lef4 was unaffected by 5/25 inhibitors, all five of which were also inactive versus MimiCE and CthTTM.

The classes of compounds that we highlight as TbCet1 inhibitors are not new to the world of medicinal chemistry or pharmacology. Diverse bioactivities of potential therapeutic value (e.g., antioxidant, anti-inflammatory, antitumor, and antimicrobial activities) have been described for caffeoylquinic acids (38), rosmarinic acid (39), pentagalloylglucose (40), and flavonoids (41, 42). A few of the TbCet1 inhibitors have been tested for cytotoxic activity against kinetoplastid protozoa. For example, 3,5-dicaffeoylquinic acid was reported to be inactive against *Leishmania amazonensis* and *Trypanosoma cruzi* amastigotes (43). However, a mixture of 3,4-, 3,5-, and 4,5-dicaffeoylquinic acids was active against *T. cruzi* trypomastigotes (44).

We conducted preliminary studies on the effects of tetracaffeoylquinic acid and rosmarinic acid on cultured procyclic *T. brucei* and found that exposure to 10 µM tetracaffeoylquinic acid or 10 µM rosmarinic acid had no effect on cell proliferation (data not shown). The lack of antitrypanosomal activity of tetracaffeoylquinic acid and rosmarinic acid could arise from either poor cellular uptake of the compounds, rapid efflux of the compounds, or their rapid intracellular catabolism.

Critical insights into the basis for TbCet1 inhibition by the various classes of phenolic compounds would be afforded by a crystal structure of RTPase-inhibitor complexes. However, our strenuous efforts to crystallize TbCet1, as apoenzyme, or in complex with Mn^2+^ and/or ATP, or as inhibitor complexes with tetracaffeoylquinic acid or rosmarinic acid, have not met with success. Absent an atomic structure, we speculate that small-molecule inhibition of TbCet1 (or other TTM enzymes) could entail plugging of the entrance of the triphosphate tunnel to prevent ingress of NTP or triphosphate-terminated RNA to the active site within the tunnel cavity.

## MATERIALS AND METHODS

### Inducible RNAi knockdown of TbCet1.

TbCet1 was depleted in *T. brucei* by using a tetracycline-inducible RNAi system as described previously (45). A 352-bp DNA fragment of the *TbCET1* open reading frame (from nucleotides 19 to 371) was PCR amplified and inserted into the p2T7-177 vector (46), wherein expression of double-stranded RNA is under the control of opposing tetracycline-inducible bacteriophage T7 promoters. The RNAi construct was electroporated into procyclic *T. brucei* 29.13 cells, which constitutively express T7 RNA polymerase and Tet repressor (47). The procyclic cell line was cultivated in SDM 79 medium supplemented with 5% fetal bovine serum, 50 µg/ml hygromycin B, and 15 µg/ml G418, and the TbCet1-RNAi transfectants were selected with the same medium plus 2.5 µg/ml phleomycin. To gauge the effects of inducible RNAi knockdown of TbCet1 on cell proliferation, equal aliquots of the transfectants were inoculated into medium containing 1.0 µg/ml tetracycline (+Tet) to induce TbCet1 dsRNA production and into a parallel control culture lacking tetracycline (−Tet). Cell density over a 14-day period was monitored by microscopy and was maintained between 1 × 10^6^ and 1 × 10^7^ cells/ml by dilution into fresh medium. Growth curves (Fig. 1A) display on the *y* axis the log of the direct cell count multiplied by the dilution factor.

Cellular levels of TbCet1 during RNAi induction were gauged by Western blotting as follows. Aliquots (30 µg of protein) of whole-cell lysates of TbCet1-RNAi transfectants harvested prior to addition of tetracycline (day 0) and after 1, 2, 6, 10, or 14 days of exposure to tetracycline were resolved by 12% SDS-PAGE. The polypeptides were transferred to a polyvinylidene difluoride membrane. TbCet1 was detected by incubating the membrane in a 1:500 dilution of affinity-purified rabbit polyclonal serum raised against recombinant TbCet1 protein (at Pocono Rabbit Farm and Laboratory, PA), followed by a 1:5,000 dilution of horseradish peroxidase-conjugated secondary antibody. Immune complexes were visualized by using an enhanced chemiluminescence (ECL) kit from Pierce. The intensity of the immunoreactive TbCet1 polypeptide was determined by scanning the autoradiogram with a Kodak Gel Logic 100 system and quantifying the signal intensity with molecular imaging software.

### Recombinant TTM proteins.

Recombinant full-length *T. brucei* RTPase TbCet1 (11, 12), the N-terminal RTPase domain (amino acids [aa] 1 to 237) of mimivirus capping enzyme (MimiCE) (18), the N-terminal RTPase domain (aa 1 to 236) of AcNPV Lef4 capping enzyme (21), the biologically active C-terminal catalytic domain (aa 241 to 539) of *S. cerevisiae* RTPase (SceCet1) (4), and full-length *C. thermocellum* TMM (CthTTM) (34) were produced in *Escherichia coli* and purified as described in the publications cited. TbCet1, MimiCE, Lef4, and CthTTM were produced as N-terminal HisSmt3-tagged fusions, and SceCet1 was produced as an N-terminal His-tagged fusion. After nickel-affinity purification of the tagged proteins from soluble bacterial lysates, the HisSmt3 tags were removed by the Smt3 protease Ulp1, and the tag-free recombinant proteins were recovered in the flowthrough fraction during a second round of nickel-affinity chromatography. The tag-free enzymes and His-tagged SceCet1 were further purified by gel filtration through a HiPrep Sephacryl S300 size exclusion column (GE Healthcare) equilibrated in buffer C (20 mM Tris-HCl, pH 8.0, 100 mM NaCl, 10% glycerol, 0 to 5 mM dithiothreitol [DTT]). Peak fractions were pooled and adjusted to attain solutions of 10 µM TbCet1, 35 µM MimiCE, 42 µM SceCet1, 500 µM CthTTM, and 7 µM Lef4. The proteins were stored at −80°C in single-use aliquots.

### HTS assay for inhibitors of TbCet1 ATPase.

The high-throughput screening (HTS) assay was based on the detection of P_i_ release from ATP via the use of malachite green reagent. P_i_ is quantified by measuring the *A*_630_ of a chromophore complex formed by malachite green and phosphomolybdate. For HTS procedures, 1 µl of test compound in 10% dimethyl sulfoxide (DMSO) was predispensed into flat-bottomed transparent 384-well plates to which was added 4 µl of ATPase master mix containing the following reagents to attain the final concentrations indicated: 50 mM Tris-HCl (pH 8.0), 20 mM MgCl_2_, 2 mM DTT, 4 mM MnCl_2_, and 2 mM ATP. Reactions were initiated with the addition of 5 µl of a solution containing 50 mM Tris-HCl (pH 8.0), 28.6 nM TbCet1, 0.02 mg/ml bovine serum albumin, 2 mM DTT, 10% glycerol, 0.005% Tween 80. “No-inhibitor” control reaction mixtures received 1 µl of 10% DMSO alone in lieu of test compound. “Complete-inhibition” control reaction mixtures received 1 µl of 400 mM EDTA in 10% DMSO. The plates were covered and incubated for 1 h at room temperature. The reactions were then quenched with 40 µl of malachite green solution, made by mixing 1 volume of 0.125% (wt/vol) malachite green in 2 M H_2_SO_4_ with 4 volumes of 0.47% (wt/vol) ammonium molybdate in 0.055% (vol/vol) Tween 20. The quenched plates were incubated for 1 h at room temperature and then scanned to quantify *A*_630_ for each reaction well. *Z*′ for the assay was gauged by comparing three 384-well plates of no-inhibitor control reactions with three 384-well plates of complete-inhibition control reactions. The effect of any test compound on TbCet1 was quantified by comparison of its *A*_630_ value for P_i_ release with those of the no-inhibitor (0% inhibition) and complete-inhibition (100% inhibition) controls. IC_50_s in the HTS assay format were determined by testing a series of 12 serial 2-fold dilutions of the test compound in 10% DMSO.

### TLC assay for ^32^P_i_ release from [γ-^32^P]ATP.

Reaction mixtures (20 µl) contained 100 mM Tris-HCl, pH 8.0, 10 mM NaCl, 1% (vol/vol) glycerol, 2 mM MnCl_2_, 200 µM [γ-^32^P]ATP, 5% DMSO, TTM enzymes (either 2.2 nM TbCet1, 7 nM MimiCE, 25 nM SceCet1, 50 nM Lef4, or 500 nM CthTTM), and inhibitors as specified. The inhibitors were serially diluted in DMSO, and 1 µl was included in each ATPase reaction mixture. The reactions were initiated by addition of enzyme. After incubation for 10 min at 37°C (for TbCet1, MimiCE, Lef4, and CthTTM) or 30°C (for SceCet1), the reactions were quenched by adjustment to 1 M formic acid. Aliquots of the mixtures were applied to polyethyleneimine cellulose TLC plates and analyzed by ascending TLC with 200 mM ammonium sulfate as the mobile phase. Radiolabeled ATP and P_i_ were visualized and quantified by scanning the TLC plate with a Fuji imager. The concentrations of input enzyme were chosen so as to attain hydrolysis of ~30% of the input ATP substrate in the “no-inhibitor” control reaction mixtures containing 5% DMSO. The extents of ATP hydrolysis were plotted versus inhibitor concentration after normalization to the “no-inhibitor” control values. IC_50_s were obtained using the “dose-response” curve fitting algorithms in Prism.
